# Probiotic Mixture Containing *Lactobacillus helveticus*, *Bifidobacterium longum* and *Lactiplantibacillus plantarum* Affects Brain Responses Toward an Emotional Task in Healthy Subjects: A Randomized Clinical Trial

**DOI:** 10.3389/fnut.2022.827182

**Published:** 2022-04-29

**Authors:** Julia Rode, Hanna M. T. Edebol Carlman, Julia König, Dirk Repsilber, Ashley N. Hutchinson, Per Thunberg, Pernilla Andersson, Jonas Persson, Andrey Kiselev, Lori Lathrop Stern, Benita Salomon, Ahmed Abdulilah Mohammed, Jennifer S. Labus, Robert J. Brummer

**Affiliations:** ^1^Nutrition-Gut-Brain Interactions Research Center, Faculty of Medicine and Health, School of Medical Sciences, Örebro University, Örebro, Sweden; ^2^Department of Radiology and Medical Physics, Faculty of Medicine and Health, School of Medical Sciences, Örebro University, Örebro, Sweden; ^3^Center for Lifespan Developmental Research (LEADER), Faculty of Humanities and Social Sciences, School of Law, Psychology and Social Work, Örebro University, Örebro, Sweden; ^4^Center for Applied Autonomous Sensor Systems, Faculty for Business, Science and Engineering, School of Natural Science and Technology, Örebro University, Örebro, Sweden; ^5^Global Medical Innovation, Pfizer Consumer Healthcare, Madison, NJ, United States; ^6^Integrative Bioinformatics and Biostatistics Core, Oppenheimer Center for Neurobiology of Stress and Resilience, UCLA Vatche and Tamar Manoukian Division of Digestive Diseases, David Geffen School of Medicine at UCLA, Los Angeles, CA, United States

**Keywords:** probiotics, functional magnetic resonance imaging (fMRI), brain activity, functional connectivity, gut-brain axis, task-related, gut microbiota, emotional attention task (EAT)

## Abstract

**Background:**

Evidence from preclinical studies suggests that probiotics affect brain function *via* the microbiome-gut-brain axis, but evidence in humans remains limited.

**Objective:**

The present proof-of-concept study investigated if a probiotic product containing a mixture of *Bifidobacterium longum R0175*, *Lactobacillus helveticus R0052* and *Lactiplantibacillus plantarum R1012* (in total 3 × 10^9^ CFU/day) affected functional brain responses in healthy subjects during an emotional attention task.

**Design:**

In this double-blinded, randomized, placebo-controlled crossover study (Clinicaltrials.gov, NCT03615651), 22 healthy subjects (24.2 ± 3.4 years, 6 males/16 females) were exposed to a probiotic intervention and a placebo for 4 weeks each, separated by a 4-week washout period. Subjects underwent functional magnetic resonance imaging while performing an emotional attention task after each intervention period. Differential brain activity and functional connectivity were assessed.

**Results:**

Altered brain responses were observed in brain regions implicated in emotional, cognitive and face processing. Increased activation in the orbitofrontal cortex, a region that receives extensive sensory input and in turn projects to regions implicated in emotional processing, was found after probiotic intervention compared to placebo using a cluster-based analysis of functionally defined areas. Significantly reduced task-related functional connectivity was observed after the probiotic intervention compared to placebo. Fecal microbiota composition was not majorly affected by probiotic intervention.

**Conclusion:**

The probiotic intervention resulted in subtly altered brain activity and functional connectivity in healthy subjects performing an emotional task without major effects on the fecal microbiota composition. This indicates that the probiotic effects occurred *via* microbe-host interactions on other levels. Further analysis of signaling molecules could give possible insights into the modes of action of the probiotic intervention on the gut-brain axis in general and brain function specifically. The presented findings further support the growing consensus that probiotic supplementation influences brain function and emotional regulation, even in healthy subjects. Future studies including patients with altered emotional processing, such as anxiety or depression symptoms are of great interest.

**Clinical Trial Registration:**

[http://clinicaltrials.gov/], identifier [NCT03615651].

## Introduction

Increasing data suggests that the bidirectional communication between the gut microbiota and the central nervous system, which is also known as the microbiome-gut-brain axis, plays a role in the development and function of the brain and, hence, may even affect behavior (1). The gut microbiota can be modulated in various ways including the intake of probiotic bacteria which typically have transient effects, gradually declining after the consumption is ended (2). Probiotics are defined as living microorganisms which, when consumed in adequate amounts, confer a health benefit to the host (3). Probiotics act *via* direct or indirect microbe-host interactions. They are speculated to affect brain function *via* the gut-brain axis, for example *via* neuronal, endocrine or immunomodulatory pathways [reviewed in (1)]. While a multitude of studies has been performed in animal models, evidence of the effects of probiotic ingestion on brain activity and the microbiome-gut-brain axis in humans remains very limited. Only a handful of clinical studies indicate that probiotic consumption might impact cognitive function and functional brain activity in general and during cognitive and emotional tasks specifically (4–9).

For instance, brain activity, in regions implicated in emotional regulation, was modulated during the Emotional Attention Task in healthy women after a daily 4-week intervention with a fermented milk product containing the four probiotic strains *Bifidobacterium animalis subsp lactis*, *Streptococcus thermophilus*, *Lactobacillus bulgaricus* and *Lactococcus lactis subsp lactis* (4). In an additional study, altered brain activity during emotional and cognitive tasks was observed after a 4-week intervention with a multi-strain probiotic containing nine bacterial strains belonging to *Lactobacillus*, *Lactococcus* and *Bifidobacterium* species (8). However, another study using the same probiotic formulation as Bagga et al. (8) could not observe any effects on task-related brain function (10). Hence, evidence of probiotic effects on brain function during emotional tasks in humans is limited and inconclusive, and further studies are needed. Probiotic effects are known to be species and strain specific and translation from preclinical to clinical studies is not always successful (11, 12). Thus, investigation of effects of a variety of probiotic strains is important. Different approaches will help to understand which probiotics might be beneficial for which outcomes and under which circumstances, hence, improving translatability.

The present proof-of-concept study investigated if a probiotic product containing *Bifidobacterium longum* R0175, *Lactobacillus helveticus* R0052 and *Lactiplantibacillus plantarum* R1012 (formerly known as *Lactobacillus plantarum*) affected functional brain responses involved in emotional regulation in healthy subjects. To investigate this, subjects were examined with functional magnetic resonance imaging (fMRI) while performing the Emotional Attention Task [EAT; (4)]. We hypothesized that the probiotic intake compared to the placebo would dampen emotional reactivity in limbic regions as well as in regions of the prefrontal and orbitofrontal cortices. In addition, to assess potential modes of action underlying the changes in brain activity, gut microbiota composition was also analyzed.

## Materials and Methods

### Design

A randomized double-blinded placebo-controlled crossover design with 22 healthy subjects was adopted. The primary endpoint was to detect the effect of the probiotic intervention on differences in brain response patterns during the EAT paradigm. Exploratory outcome parameters included, amongst others, the effect of the probiotic intervention on markers of importance for the microbiome-gut-brain axis as well as microbiota composition in fecal samples.

**FIGURE 1:**
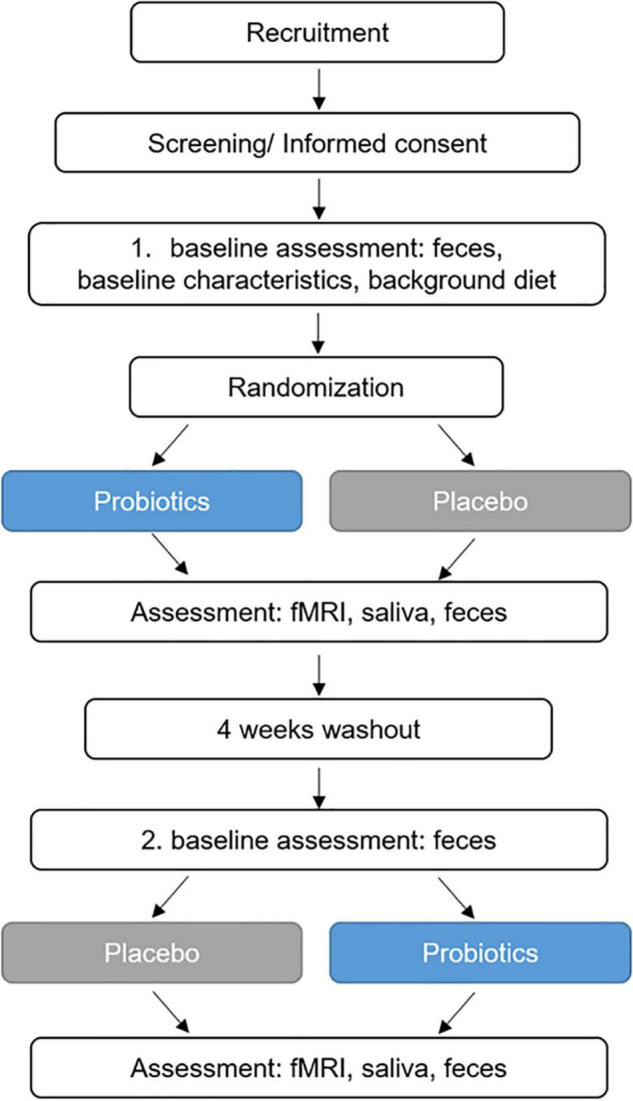
Study design. fMRI, functional magnetic resonance imaging.

After baseline assessments of mental health, study participants underwent a 4-week intervention with the study product or the placebo, followed by a 4-week washout period and a subsequent 4-week intervention period (placebo or study product, respectively). The EAT was performed during fMRI measurements after each intervention period. fMRI measurements at baseline were not performed in order to avoid bias due to potential learning effects. At several time points during the fMRI investigations, saliva samples were collected for cortisol analysis. Before and after (up to maximal 4 days before the fMRI examinations) the first and second intervention period, respectively, fecal samples were collected. Figure 1 presents the design and key events in the study.

### Subjects

Sample size calculation was performed considering exclusively the primary outcome as the standard procedure in the field. The primary outcome was to investigate probiotic intake associated alterations in brain response patterns during the EAT paradigm, taking into account the crossover design of our study. Hereto, the two interventions (probiotics vs. placebo) should be compared within the same subject. Power calculations were, therefore, based on a paired *t*-test to enable us to detect a change in functional connectivity as large as the observed standard deviation for a single functional connection (measured as partial correlation) between two given brain regions, i.e., aiming to be able to detect an effect size of *Cohen*′*sdz* = 1. We aimed at a power of 80%, a 95% confidence interval (i.e., significance level at 5%), and Bonferroni correction for the multiple brain regions analyzed (5 defined brain regions yield 10 pairwise interactions). Considering these constraints, a minimum of 18 subjects would be required to demonstrate, for example, a mean difference (denoted as beta values in CONN18.b) of = *u*_1_−*u*_2_ = 0.3(*sd* = 0.3*Cohen*′*sdz* = 1), i.e., a difference between partial correlations *u*_1_ = 0.3 and *u*_2_ = 0.6, at α = 0.05 and for a maximum dropout rate of 20%. The above explained sample size calculations were performed based on general assumptions, since prior information on expected and relevant effect sizes for the combination of treatment and outcome investigated in the presented study did not exist.

Volunteers were recruited by advertisement at Örebro University in 2018. Those interested received an information letter and were prescreened *via* telephone. After an additional personal information meeting, participants fulfilling the inclusion criteria (aged 18–65 years, males/females, signed informed consent) were included by the principal investigator if none of the exclusion criteria in Table 1 was met. A participant flow chart can be found in Supplementary Figure 1. The order of administration of the intervention (probiotics or placebo first) was done using a computerized randomization list, and block randomization with a random block size of six and four was applied. Half of the study group received the probiotics first and the placebo second, which was *vice versa* for the other half of the study group. The assignment of administration order to the two groups and labeling of the sachets with subject ID and number of intervention period was done by a university staff member not involved in the study. The randomization key has was controlled by this person and not revealed until analysis of the primary outcome parameter was finished. The study was blinded for participants and study staff.

**TABLE 1:** Exclusion criteria.

1. Concurrent or recent (<12 weeks) treatment with drugs affecting intestinal function or mood, e.g., antidepressants or antibiotics
2. Concurrent or recent (<4 weeks) use of nutritional supplements or herb products affecting intestinal function or mood (e.g., aloe vera, St. John’s Wort, fibers, prebiotics and probiotics)
3. Diagnosis of major psychiatric or somatic disease
4. Abuse of alcohol or drugs
5. Recent (<4 weeks) intake of proton pump inhibitors (e.g., omeprazol)
6. Asthma
7. Cardiovascular diseases
8. Epilepsy
9. Renal failure
10. Cerebral bleeding or history of cerebral bleeding
11. Allergy to latex 12. Pregnancy (assessed by urine test) or breastfeeding 13. Claustrophobia 14. Smoking or using tobacco including snuff 15. Inability to maintain exercise routine and dietary pattern during the study 16. Consumption of more than six cups of coffee/caffeine-containing beverages per day 17. Professional athlete 18. Any contraindication to an MRI (e.g., medical implant or device not compliant with MRI) 19. Recent (<3 months) regular intake of systemic corticosteroids and anti-inflammatory medication (including non-steroidal anti-inflammatory drugs) 20. Known allergy to milk or soy 21. Any other reason the investigator felt the subject was not suitable for participation in the study

*MRI, magnetic resonance imaging.*

Background diet was registered with a 3-day food diary (13) at baseline in which participants registered all food and drink consumed over the course of two weekdays and 1 day on the weekend. Physical activity was recorded during 1 week before the start and during the last week of each intervention period by Actigraphy (Actiwatch spectrum-pro and Actiware 6.0 software, Philips Respironics, United States).

Participants were instructed not to make any major dietary, medication or lifestyle changes without notifying the study team.

### Study Intervention

The probiotic product, commercially available in Italy (named Puraflor, GSK Consumer Healthcare, Italy), was manufactured by a third party manufacturer, SIIT S.r.l. (Italy), and contained a food grade approved combination of the three probiotic strains: *Lactobacillus helveticus* R0052 (CNCM-I-1722; 2 × 10^9^ colony forming units (CFU) per day at the end of shelf-life, i.e., number of viable bacteria), *Lactiplantibacillus plantarum* R1012 (CNCM-I-3736; 8 × 10^8^ CFU), and *Bifidobacterium longum* R0175 (CNCM-I-3470; 7 × 10^7^ CFU), in addition to inulin, zinc, magnesium, potassium, glutathione and lactoferrin (exact composition can be found in Supplementary Table 1). The probiotic strains were present at a minimum of 3 × 10^9^ CFU (at end of shelf-life) per 3 g daily powder sachet. Subjects recorded the daily intake of the probiotics and returned any unused sachets at the end of each intervention period for purposes of measuring compliance.

The placebo product was a 2 g powder sachet formulated to have a similar appearance and taste as the probiotic product, but without the active ingredients (Supplementary Table 1). Study participants were instructed to consume one sachet per day immediately after dissolving it in a glass of water at room temperature together with breakfast.

### Functional Magnetic Resonance Imaging Protocol

fMRI examinations were conducted at the end of the 4 weeks of probiotic and placebo intervention, respectively. The imaging protocol included an initial 4.5-min structural scan, followed by a 5-min resting scan (eyes closed) (not included in the current analysis), and then the EAT paradigm. All fMRI acquisitions were performed with the same protocol, implying an equal sequential order for all acquisitions. A 3.0T MR system (Discovery 750w, GE Medical Systems, United States) and a 32 channel fMRI head coil were used for all MR examinations. The structural scan (T1-weighted IR-prepared fast spoiled gradient recalled echo, “BRAVO”) had the following parameters applied; TR/TE = 8.6/3.3 ms, acquired voxel size of 0.9 × 0.9 × 1.2 mm^3^, while the fMRI acquisitions were based on a gradient echo EPI pulse sequence using the following parameters: TR/TE = 2500/35 ms, slice thickness 3.6 mm, no slice gap, in-plane resolution of 3.75 × 3.75 × 3.6 mm^3^ and a reduction factor (ASSET) of 2.

Saliva samples were collected at each fMRI visit before and after the EAT paradigm in order to measure salivary cortisol concentrations as a marker of stress. Subjects were not allowed to perform physical exercise the same day as the fMRI assessment and were allowed to drink a maximum of one cup of coffee or tea in the morning. Subjects were allowed to follow their individual routines (e.g., time of wake up, morning activities, breakfast), but were instructed to have the same routines on both days of the fMRI examinations in order to avoid any confounding effects on the measured parameters. Furthermore, the two fMRI examinations of each subject were performed at the same time of the day.

#### The Emotional Attention Task

The EAT paradigm (4) consisted of an experimental condition named Match Emotions (ME) using faces with emotional expressions (fear or anger) [from Nim Tottenham picture data bank (14)] and a control condition named Match Shapes (MS) (ovals or circles). The entire task took 8 min. Both conditions were performed in a block design of four blocks in total in the following order: MS - ME - ME - MS. Each block consisted of 20 trials, presented for 5 s with no interstimulus interval. Then, either the instructions “match emotions” or “match shapes” were presented for 10 s followed by 10 s of crosshair fixation before the next block started. During each trial, the subject was instructed to identify the emotion/shape at the top of the screen and match it with the corresponding one at the bottom of the screen. Answers were given by handheld response buttons.

A scheme of the EAT paradigm can be found in Supplementary Figure 2.

##### Analysis of Behavioral Data During Emotional Attention Task

The number of total, correct and incorrect answers given during EAT were assessed in order to control if subjects were engaged in the task. 20 answers (one per trial) were expected per block. Intervention effects were analyzed by Wilcoxon matched-pairs signed rank test (GraphPad Prism 8, GraphPad Software Inc., United States) as data was non-normally distributed (determined by Shapiro–Wilk Test) as most subjects answered correctly. Data is presented as median and interquartile range (IQR 25–75).

### Functional Magnetic Resonance Imaging Data Analysis

#### Task-Related Functional Magnetic Resonance Imaging Activity Analysis

##### Preprocessing

SPM12 (Statistical Parametric Mapping, The Wellcome Centre for Human Neuroimaging, UCL Queen Square Institute of Neurology, United Kingdom) was used for data preprocessing (Matlab 9.3 R2017b, The Mathworks Inc., United States). Default settings were used if not stated otherwise. Functional images were co-registered with the structural high-resolution scan and normalized (warped) into Montreal Neurological Institute (MNI)-space and resampled to a voxel size of 3 × 3 × 3 mm^3^ before smoothing using a Gaussian kernel (smoothing width set to 6 mm). The series of ready-pre-processed fMRI NIfTI-files for each individual and visit was then used as input for the statistical analysis as described below. A detailed list of the preprocessing steps can be found in the Supplementary Information.

##### Task-Related Functional Magnetic Resonance Imaging Activity Analysis

First, we *a priori* selected regions based on their relevance in the literature and examined brain responses during the EAT paradigm after the placebo intervention in order to validate our setup of the paradigm and to examine regions whose activation changed in response to the task itself. Then, we selected those regions that were involved in the task which overlapped with the *a priori* literature-based selection, as regions of interest (ROIs), for the intervention contrast. Thereafter, we compared brain activity between the probiotic and placebo intervention. Brain response to the EAT was measured as a contrast of ME − MS. The general linear model was used to model blood-oxygen-level-dependent (BOLD) timeseries as a function of explanatory variables or regressors. Statistical analyses were performed in R 4.0.0 (15), based on the expected BOLD response for the task indicator function given by the EAT design, as a convolution with the hemodynamic response function (HRF), proposed in package “fmri” (16, 17).

###### Brain Responses to Emotional Attention Task Paradigm After Placebo Condition

To determine brain responses to the EAT paradigm after the placebo intervention at the voxel/region/cluster level we specified the following linear model.

Model definition:

Equation 1:


$$y_{i}=\beta_{0}+\beta_{1}*t+\beta_{2}*I\,N\,S\,T\,R+\beta_{3}*F\,I\,X+\beta_{4}*\left(M\,E+M\,S\right)+\beta_{5}*\left(M\,E-M\,S\right)+e\,r\,r$$

Where β_0_ denoted a common intercept, β_1_ the coefficient of a linear drift effect for normalization, β_2_ a component in place for those times in the paradigm when instructions were given (with INSTR as indicator variable) and β_3_ a component to fit values for the times when a crosshair should be focused on (with FIX being the indicator variable). β_4_ denoted the coefficient of an average component of the ME and the MS part of the paradigm, whereas β_5_ denoted the coefficient of a component specifying the differences between these two blocks (ME − MS) and thus reflected the pure emotional response to the EAT paradigm. Both effects, ME + MS and ME − MS are needed in the model to reflect all possible activations due to both parts of the paradigm, ME and MS. ME and MS were themselves specified as standard models of the BOLD HRF, with default parameters as proposed by Worsley et al. (16) and by Polzehl and Tabelow (17). Results from this analysis were compared to results from an earlier publication (4) to demonstrate the reliability of the task. The model was analyzed as classical linear model (employing R-function lm), without taking any individual effects into account (no random effects modeled). Results are, hence, expected to be conservative with regard to reporting significant voxel effects which would be significant even on a pure group-level comparison. In contrast, for the analysis of intervention effects, individual effects (random effects) were modeled to enhance power, as detailed below.

###### Selection of Regions of Interest

We used a stepwise process to define ROIs for the comparison of the probiotic and the placebo intervention in terms of brain responses to the EAT paradigm. First, a collection of regions (*n* = 30) was *a priori* selected based on relevant peer-reviewed research investigating the effect of probiotic intake on emotional attention (4). Furthermore, as the EAT paradigm involves the processing of emotional faces, we were interested in regions implicated in stress and anxiety (18, 19) and emotional processing (20, 21). Therefore, we selected regions such as the amygdala, hippocampus, insula, nucleus accumbens, cingulate cortex, and prefrontal cortex (Supplementary Table 2).

Second, we conducted an additional selection approach to examine activity within sets of voxels that showed a positive difference subtracting the BOLD signals of MS from ME (ME − MS) with regards to brain activity after the placebo intervention. Then, we used these sets of coherent voxels to further model differences in activity in response to intervention using a cluster-based approach focusing on functionally defined regions. Finally, we examined the overlap of the literature-based pre-defined regions and the sets of voxels whose activation changed in response to the task (the EAT paradigm) to choose the final set of ROIs.

###### Definition of Clusters

*P*-values of individual voxels’ linear models and their ME-MS effects were assessed applying Bonferroni correction for level of significance and negative log10 (p)-values saved as 3D-NIfTI files. Neighboring voxels with altered signal intensities, as defined by a significant ME − MS effect in the voxel wise analysis (significant β_4_ in the model above, with nominal *p*-value < 10^–50^) were defined as clusters as detected by density-based spatial clustering (22, 23) used with a critical epsilon neighborhood set to eps = 1.5 and a minimum number of points set to minPts = 10. For annotation of brain regions, these *p*-value brain volumes in MNI space were displayed using brainnetome atlas (BNA) (24). Using FSLview, the islands of significant *p*-values were examined visually and located within their functional BNA atlas regions. Clusters spanning over several BNA regions were split into several sub-clusters, each covering only one BNA region. In total 55 sub-clusters were defined. A limited number (*n* = 10) of those sub-clusters overlapped with *a priori* literature-based selected ROIs (Supplementary Table 3 and Supplementary Figure 3). For each cluster, a peak coordinate was defined as the voxel with the most significant difference ME − MS, i.e., showing a specific response to the emotion matching part of the paradigm. fMRI activity profiles for sub-clusters were computed as averages of the composing voxels prior to the analysis of intervention effects. Accordingly, signal intensities of all composing voxels of individual sub-clusters were averaged for each brain volume separately, thus keeping the time course of the signal.

###### Analysis of Intervention Effects

For identifying intervention effects in the crossover design of the current study, a mixed effects model with fixed effects as in the model above (Equation 1) plus a treatment effect and an interaction of treatment and ME − MS effect was built:

Equation 2:


$$y_{i}=\beta_{0}+\beta_{1}*t+\beta_{2}*I\,N\,S\,T\,R+\beta_{3}*F\,I\,X+\beta_{4}*\left(M\,E+M\,S\right)+\beta_{5}*\left(M\,E-M\,S\right)+\beta_{6}*T\,R\,E\,A\,T+\beta_{7}*T\,R\,E\,A\,T*\left(M\,E-M\,S\right)+e\,r\,r$$

In addition, random effects for the subject-specific treatment effects (β_6_), for the combined ME and MS effect (β_4_), as well as for the ME − MS differential effect (β_5_), and for its interaction with the treatment effect (β_7_), were analyzed to account for repeated data from the same individual.

The script of the fMRI activity analysis can be found in the Supplementary Information.

#### Task-Related Functional Magnetic Resonance Imaging Connectivity Analysis

Functional connectivity analysis was performed in CONN connectivity toolbox version 18.b standalone (25) using Matlab 9.5 R2018b. The CONN default preprocessing pipeline was used for data preprocessing with conservative settings (95th percentile) for ART-based outlier detection for scrubbing. Functional and structural scans were realigned, slice-time corrected, segmented and normalized into MNI-space before smoothing using a Gaussian kernel (smoothing width set to 8 mm). Denoising was performed with sequential regression (RegBP), a band-pass filter of 0.008–0.09 Hz, linear detrending and no despiking. The data of two subjects was excluded due to technical reasons. The interventions were compared with respect to the differential connectivity evoked by the EAT paradigm. A ROI-to-ROI analysis (bivariate correlation, HRF weighting) of sub-clusters showing significant activation during EAT after the placebo intervention (described above) was performed. As initial cluster definition was based on the contrast between the ME and MS condition, connectivity analysis could be performed on a comparison of the ME conditions after both of the interventions. A two-sided, seed-level correction using Benjamini–Hochberg’s false discovery rate (FDR) < 0.05 was used as threshold for the ROI-to-ROI connections for a matrix of 55 × 55 clusters.

#### Visualization of Functional Magnetic Resonance Imaging Data

The software multi-image analysis GUI (MANGO, Research Imaging Institute, University of Texas Health Science Center, United States) was used to visualize functional images. During analyses, all images were validated in FSL and Mango in order to avoid potential discrepancies.

### Saliva Samples Collection and Analysis

Saliva samples were collected for 1 min using Salivette collection tubes (Sarstedt, Germany) for measurement of cortisol during the fMRI examinations: upon arrival at the study center, just before entering the fMRI scanner, before and after the EAT paradigm. The Salivette collection tubes were kept at 4°C, centrifuged at 1,000 *g* for 1 min, transferred to Eppendorf tubes (Eppendorf, Germany), immediately frozen in −20°C and subsequently stored at −80°C until analysis. For biochemical analysis, samples were thawed and salivary cortisol concentrations measured in duplicates using a chemiluminescent immunoassay with high sensitivity and a minimal detection of 0.44 nmol/L (IBL, Germany) at DresdenLab Service GmbH (Dresden, Germany). Intra- and inter-assay coefficients of variation were below 8%. The baseline-corrected effects of probiotics and placebo, respectively, on salivary cortisol concentrations after the EAT paradigm were analyzed using paired samples *t*-tests (GraphPad Prism 8) as data was normally distributed (assessed by Shapiro–Wilk test). For baseline-correction purposes, saliva samples obtained just before the subject performed the EAT paradigm (thus after the anatomical and resting state scan) was used. The results of one subject were excluded from analysis as outlier with the lowest value of this subject being more than 3.5 standard deviations higher than the average value of all values from all other subjects. Data is presented as median and IQR.

### Subjective Ratings

After the second fMRI occasion, subjects were asked to rate their subjective stress perception during the fMRI itself and during the EAT paradigm at both fMRI visits on visual analogous scales (VAS). Differences were analyzed by Wilcoxon matched-pairs signed rank test (GraphPad Prism 8) as data was non-normally distributed (assessed by Shapiro–Wilk Test). Data is presented as median and IQR.

### Microbiota Analysis

Fecal samples were collected at home before and after probiotic and placebo intervention, respectively. At home, samples were immediately stored frozen in DNA/RNA Shield (ZymoResearch, United States) and after a maximum of 2 days transferred in cooling transport systems (Sarstedt, Germany) to the study unit where samples were stored at −80°C until analysis. The DNA was mechanically extracted from the fecal samples including a bead-beating step at 1,600 rpm as described by Hugerth et al. (26) at the Centre for Translational Microbiome Research (CTMR) (Karolinska Institutet, Stockholm, Sweden). Microbial composition was analyzed by 16S rRNA-based Next Generation Sequencing targeting the V3-V4 variable region according to Hugerth et al. (26) at CTMR. In short, template generation and sequencing were performed using the following: Ion PGM OT2 400 Kit; Ion PGM Sequencing Kit 400; Ion 318v2 chip (Gibco Life Technologies, United States). Analysis was performed according to manufacturer’s instructions.

The CTMR sequence processing pipeline is based on DADA2 version 1.8 (27) and implemented in Nextflow (28). The 17 and 21 bases from the 5′-ends, and 15 and 75 bases from the 3′-ends for all reads were trimmed off to remove the degenerated primer sequences and low-quality sequence tails after checking the reads’ quality profiles. In addition, PhiX sequences and reads that contained “N” or have more than two “expected errors” were removed. Then, the DADA2 denoising program was applied to the quality-filtered reads to improve the reads’ quality further. Requiring at least a 30 bases overlapping region, the paired forward and reverse reads were merged to recover the amplicon sequences and then annotated with Silva ribosomal RNA database version 128 (29).

#### Data Analysis of Microbiota Data

The statistical analysis of the microbiota data was performed using R (version 3.6.2) (15). The read counts of the amplicon sequence variants were merged by the taxonomic annotation of the respective taxonomic rank (30, 31). The read counts were then normalized for library size (30). In order to detect significant differential effects of the probiotic intervention compared to placebo on microbiota composition, univariate analyses on various taxonomic levels were performed. For the univariate analyses, taxa that were absent in more than 85% of the samples were removed. It was controlled that the removed taxa did not follow a specific pattern. Zero-inflated negative binomial regression models were used for univariate differential abundance analyses. The model was specified as follows:

Equation 3:


$$y=NB(\beta_{0}+\beta_{1}*TREAT+\beta_{2}*TIME+\beta_{3}*TREAT*TIME+err)$$

Y denoted the fitted library-size normalized taxon abundance, NB the negative-binomial link function, β_0_ the intercept, β_1_ the coefficient for the treatment effect TREAT (with two levels, “placebo” and “probiotics”), β_2_ the coefficient for the time-point effect TIME (with two levels, “before intervention” and “after intervention”), and β_3_ the coefficient of their interaction TREAT × TIME (being non-zero for the combination of “probiotics” and “after intervention”). The interaction was used to test for the post-treatment effects of the probiotic compared to the placebo intervention. Benjamini Hochberg’s method was used to correct for multiplicity (32, 33). The FDR cutoff was set to 5%. Alpha diversity was calculated using the Simpson’s and the Shannon–Weaver index (30, 34, 35). The effects of placebo and probiotic intervention (before vs. after intervention) on these diversity measures were investigated using a Wilcoxon matched-pairs signed rank test. To test for differential effects between the interventions Friedman-test was used. Multivariate analysis was performed using Bray-Curtis dissimilarity scores applied in a principal coordinate analysis (31, 35–37).

### Assessment of Multiplicity in Our Analyses

Generally, for fMRI and microbiota results, nominal *p*-values are reported for *p* < 0.05 as well as Benjamini–Hochberg’s false discovery rate (FDR) estimates with a cutoff FDR < 0.05 as well as results after Bonferroni corrections, before final conclusions.

### Ethical Statement

The study protocol was approved by the Ethical Review Board of Uppsala, Sweden (^1^ registration number: 2017/398 A and B), conducted according to Good Clinical Practice and in accordance with the Helsinki Declaration and its revisions. The study was registered at ClinicalTrials.gov (NCT03615651). Participation was voluntary and all subjects had the right to withdraw from the study at any time. Participants were paid 6,000 Swedish kronor for compensation of discomfort and time (taxable income) and reimbursement for travel costs. Any adverse event was recorded and the investigator assessed each event with regard to its severity and possible relation to the intervention.

### Data Sharing Statement

The original microbiota sequencing data presented in the study are publicly available. This data can be found here: BioProject: PRJNA789789; http://www.ncbi.nlm.nih.gov/bioproject/789789. Other original data presented in the manuscript will be made available upon request.

## Results

### Subject Characteristics

The study was completed by 22 healthy participants (16 females, 6 males) with a mean age of 24.2 ± 3.4. Subjects’ baseline characteristics are reported in Supplementary Table 4. Background diet of all subjects is presented in Supplementary Table 5. Physical activity is presented in Supplementary Table 6. No serious adverse events were reported (Supplementary Table 7). Compliance was good: All subjects consumed 90% or more of the study product (Supplementary Tables 8, 9). None of these differed significantly between the two study arms (1. probiotics and 2. placebo vs. 1. placebo and 2. probiotics).

### Engagement During the Emotional Attention Task

Subjects were adequately engaged in the task with a median of 20.0 (IQR 20.0–20.0) total, 20.0 (IQR 19.5–20.0) correct and 0.0 (IQR 0.0–0.0) incorrect answers during each block of 20 MS trials and 20.0 (IQR 19.5–20.0) total, 18.5 (IQR 17.6–19.5) correct and 1.0 (IQR 0.5–2.0) incorrect answers during each block of 20 ME trials. There was no statistical difference in terms of engagement between the interventions.

### Validation of the Paradigm Setup for the Emotional Attention Task

In order to validate if the EAT paradigm worked as previously described (4) in the presented setting, modeling was used to assess which brain regions showed significantly altered brain activity comparing the emotional condition with the control condition (ME vs. MS) after the placebo intervention. A whole-brain analysis showed Bonferroni-corrected (*p* < 0.05/246) significant increases in activity in precuneus, superior frontal gyrus, ventral caudate, superior parietal lobule, hippocampus, fusiform gyrus, middle frontal gyrus, insular gyrus and amygdala, amongst others, similar to what has been previously reported (4). These regions were used for subsequent analyses of changes in task-related activity comparing the probiotic and placebo intervention (Supplementary Table 2).

### Task-Related Brain Activity in Regions Associated With Emotional, Cognitive and Face Processing Was Affected by Probiotic Intake

**FIGURE 2:**
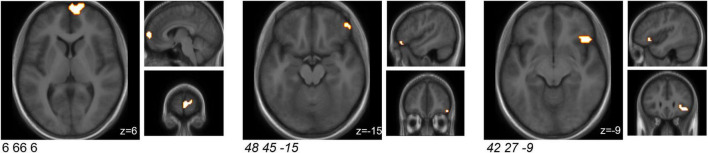
Sub-clusters that were found to be associated with significant (*p* < 0.05) changes in brain activity between both interventions (probiotic > placebo) before multiplicity correction, during the EAT paradigm. Two of these sub-clusters covered predefined ROIs (italic). Clusters are superimposed on average anatomical scans. Clusters can be identified by the coordinates of their peak (x y z). EAT, emotional attention task; ROI, region of interest.

A cluster-based analysis was performed in order to focus on functionally altered sets of voxels. Significant (*p* < 0.05) activation (without correction for multiplicity) was observed in three predefined sub-clusters when comparing the interventions with respect to the differential activity evoked by the challenge paradigm. Two of these sub-clusters were located within the predefined ROIs, both within the orbitofrontal cortex (Table 2 and Figure 2). All clusters showed an increased activation upon probiotic intervention compared to placebo.

**TABLE 2:** Sub-clusters that were found to be associated with significant (*p* < 0.05) changes in brain activity between both interventions before multiplicity correction, during the EAT paradigm.

MNI coordinates of peak (x y z)	Cluster size [mm^3^]	ME − MS in probiotic − placebo *p*-value*	ME − MS in probiotic − placebo effect size	Anatomical region
6 66 6	2,673	0.007	7.110	Medial area 10
*48 45* −*15*	*378*	*0.038*	*3.451*	*Orbital gyrus, orbital area 12/47*
42 27 −9	*1,188*	*0.041*	*1.722*	*Lateral area 12/47*

*After correction for multiple testing using Bonferroni, the changes in activation in all sub-clusters (p > 0.05/55) and the predefined ROIs (p > 0.05/10) did not remain statistically significant, nor after FDR correction. EAT, emotional attention task; FDR, false discovery rate; ME, match emotions; MNI, Montreal Neurological Institute; MS, match shapes; ROI, region of interest. Two of these sub-clusters covered predefined ROIs (italic).

Additionally, a whole-brain analysis based on anatomically-defined brain regions was performed and can be found in the Supplementary Information and Supplementary Table 10.

### Brain Connectivity During the Emotional Attention Task Was Affected by Probiotic Intake

**FIGURE 3:**
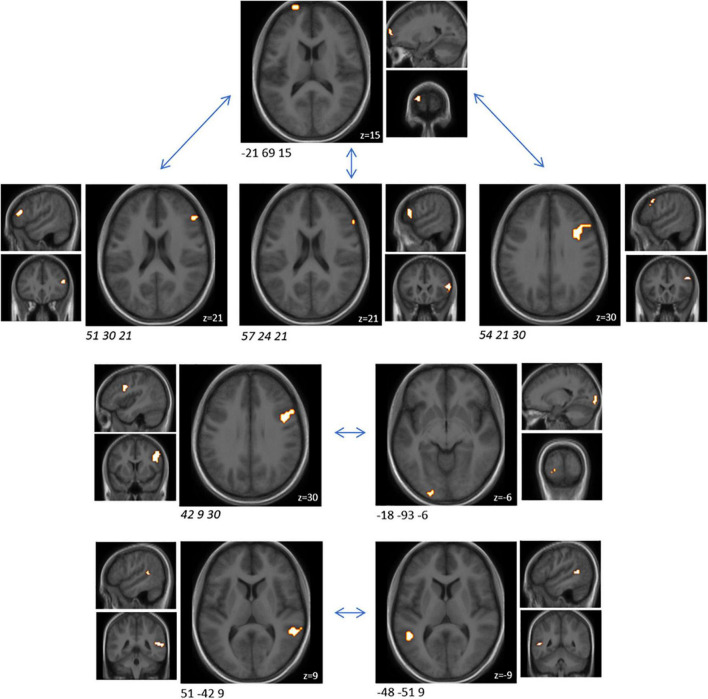
Cluster pairs that were found to be associated with significant (FDR-corrected *p* < 0.05) connectivity changes between both interventions (probiotic < placebo), during the EAT paradigm. None of these sub-clusters was among the predefined ROIs. Four of those sub-cluster belonged to the same larger cluster spanning over several BNA regions (italic). Clusters are superimposed on average anatomical scans. Clusters can be identified by the coordinates of their peak (x y z). BNA, Brainnetome atlas; EAT, emotional attention task; FDR, false discovery rate; ROI, region of interest.

In order to understand how the probiotic intervention altered task-related functional connectivity, a connectivity analysis between all sub-clusters that showed altered brain activity when comparing the emotional with the control condition was performed (i.e., ME vs. MS). Probiotic intervention resulted in significantly (FDR-corrected *p* < 0.05) decreased connectivity between several sub-clusters for the ME condition (Table 3 and Figure 3). None of these sub-clusters was among the predefined ROIs. Nevertheless, some of them were sub-clusters of larger clusters spanning partly over predefined ROIs.

**TABLE 3:** Cluster pairs that were found to be associated with significant connectivity changes between both interventions, during the EAT paradigm.

Cluster size (mm^3^)	MNI coordinates of peak (x y z)	Anatomical region	Cluster size (mm^3^)	MNI coordinates of peak (x y z)	Anatomical region	T (probiotic—placebo)	FDR
756	−21 69 15	Frontal pole	1,134	*57 24 21*	*Caudal area 45*	−4.51	0.013
*3,402*	*42 9 30*	*Caudal ventrolateral area 6*	1,107	−18 −93 −6	Occipital polar cortex	−4.18	0.027
756	−21 69 15	Frontal pole	*3,051*	*54 21 30*	*Inferior frontal junction*	−3.85	0.030
756	−21 69 15	Frontal pole	*1,107*	*51 30 21*	*Inferior frontal sulcus*	−3.46	0.047
945	51 −42 9	Caudoposterior superior temporal sulcus	594	−48 −51 9	Caudoposterior superior temporal sulcus	−3.98	0.044

*BNA, Brainnetome atlas; EAT, emotional attention task; FDR, false discovery rate; MNI, Montreal Neurological Institute; ROI, region of interest. None of these sub-clusters was among the predefined ROIs. Four of those sub-cluster belonged to the same larger cluster spanning over several BNA regions (italic).*

### Stress Induction During the Emotional Attention Task Was Not Affected by Probiotic Intake

**FIGURE 4:**
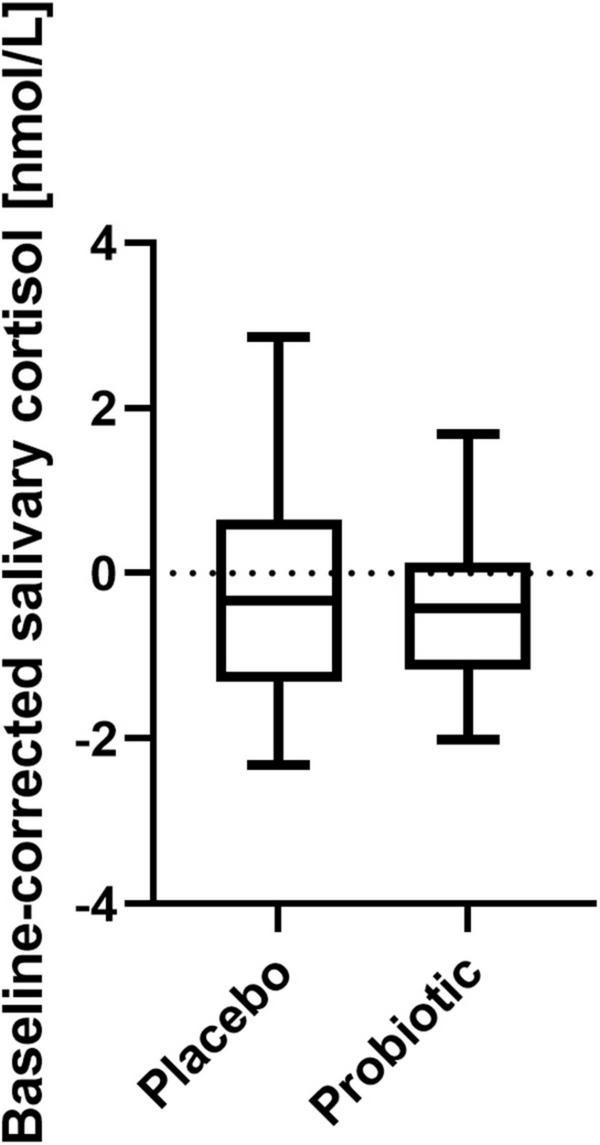
Baseline-corrected salivary cortisol concentrations after EAT after placebo and probiotic intervention. No statistically significant differences were found between both interventions. Line presents median, box presents 25th and 75th percentile, whiskers present minimum to maximum; paired *t*-test; *n* = 21. EAT, emotional attention task.

**FIGURE 5:**
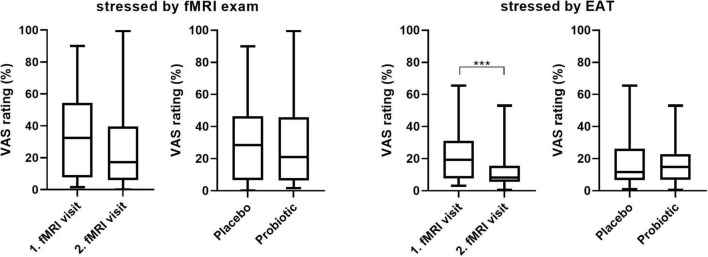
Subjective stress ratings (VAS 0-100) of the fMRI examinations itself and the EAT paradigm by visit and intervention. Wilcoxon matched-pairs signed rank test; *n* = 22; *** *p* < 0.001; line presents median, box presents 25th and 75th percentile, whiskers present minimum to maximum. EAT, emotional attention task; fMRI, functional magnetic resonance imaging; VAS, visual analogous scale.

Stress-inducing effects of the fMRI EAT paradigm were assessed by salivary cortisol and self-rated VAS. Salivary cortisol did not increase significantly in response to exposure to the EAT paradigm: Baseline-corrected salivary cortisol concentrations after EAT (Δ after EAT − before EAT) did not significantly differ between both interventions (*p* = 0.426) (Figure 4). Furthermore, salivary cortisol concentrations did not differ at any other sampling time point (upon arrival at the study center, just before entering the fMRI scanner, and before EAT) when comparing the two interventions (data not shown). In addition, based on subjective stress ratings (Figure 5) subjects felt more stressed by the fMRI examination itself than by the EAT, albeit non-significantly. There was no significant difference in subjective stress ratings for EAT between interventions (*p* = 0.744). However, participants felt significantly (*p* < 0.001) less stressed during EAT at their second fMRI occasion [median 8.3% (IQR 5.6–15.6)] compared to the first [median 19.3% (IQR 7.8–31.1)], independent of intervention. This could partly be explained by a feeling of slightly, albeit non-significant (*p* = 0.062), less stress due to the fMRI examination itself at their second fMRI occasion [median 17.3% (IQR 6.0–33.6)] compared to the first [median 32.5% (IQR 7.6–54.3)].

### The Fecal Microbiota Composition and Its Diversity Were Not Substantially Affected by Probiotic Intake

In order to assess whether the probiotic intervention affected the gut microbiota composition, 16S NGS was performed (data not shown). The treatment-time interaction of the phylogenetic order Selenomonadales was significant (FDR = 0.037), indicating differential effects of the probiotic compared to the placebo intervention. The effects of the probiotic or placebo intervention themselves (before vs. after intervention) were not significant (FDR > 0.3): The relative abundance of the order Selenomondales was non-significantly decreased after the probiotic treatment and non-significantly increased after the placebo treatment, both compared to the respective baseline. The relative abundances of no other taxa were significantly affected by probiotic or placebo intervention. The relative abundance of the species *Bifidobacterium longum* and the genera *Bifidobacteria* and *Lactobacillus*, all contained in our probiotic mixture, were slightly but not significantly increased after the probiotic intervention and decreased after the placebo intervention when compared to its respective baseline; no differential effects of the probiotic compared to the placebo intervention could be observed. Also, within-sample diversity (alpha diversity) and between-sample diversity (beta diversity) were not significantly affected by the probiotic intervention: Compared to the placebo intervention, the probiotic intervention did not evoke differential effects on diversity measured as Shannon–Weaver index (*p* = 0.569) or Simpson’s index (*p* = 0.740). Also, compared to its respective baseline, each intervention itself did not evoke differential effects on Shannon–Weaver (*p* = 0.235 for placebo, *p* = 0.503 for probiotics) or Simpson’s indices (*p* = 0.337 for placebo, *p* = 0.388 for probiotics).

## Discussion

Modulations of the human gut microbiota and subsequently the gut-brain axis, by e.g., probiotics, have been shown to affect brain activity and behavior, particularly in animal studies. This is one of only a few probiotic intervention studies in humans to investigate this matter and the first study that was able to detect differences in brain activity to the EAT paradigm after probiotic intervention in a study population of both, male and female healthy subjects. We could show that a 4-week intervention with a probiotic product containing *Bifidobacterium longum*, *Lactobacillus helveticus* and *Lactiplantibacillus plantarum* altered the brain response pattern during the EAT in our study population. Increased brain activity was observed in brain regions implicated in the processing of emotion, cognition and emotional faces when comparing the emotional (ME) to the control (MS) condition. The response pattern, measured as differences between those conditions, was altered after probiotic intervention compared to placebo, with increased activity in some brain regions and decreased connectivity between others. Subjects were adequately engaged and not particularly stressed by the task. Gut microbiota composition was only minimally altered.

During the EAT paradigm, the overall comparison of the emotional and the control condition replicated previously described changes in brain activation (4, 38). Hence, we confirmed that the paradigm worked very well in our setting and was able to evoke activation in areas related to emotional attention and processing.

Next, effects of the probiotic intervention (compared to placebo) on task-related brain activity in orbitofrontal regions (part of the prefrontal cortex) were observed—a region that projects to multiple limbic regions and is integral to emotional processing. Pictures of emotional faces are known to increase activity in a wide variety of brain regions implicated in emotional recognition and perception (38), including the amygdala, posterior hippocampus, superior temporal gyrus, insula, and anterior cingulate. Our findings support the notion that the probiotic supplementation modulates brain activity especially in regions known to regulate emotional processing. Additionally, we have observed decreased task-related functional connectivity after the probiotic intervention compared to the placebo in a number of brain regions implicated in the processing of emotion, attention and cognition. More specifically our data suggest that the specific combination of probiotic strains we applied may act to reduce connectivity of these regions in response to negative emotional stimuli, thus dampening negative emotional responses. Reduced connectivity could be a sign of higher brain efficiency, meaning that less effort is needed to fulfill the task. Even though the brain activity and functional connectivity results seem to contradict each other in the first place, they align with the novel proposal of decreased functional connectivity due to an overall increase of brain activity (39).

In the present study, we employed both—a task-related activity analysis as well as a task-related functional connectivity analysis—in order to investigate the activation of individual brain regions as well as their interplay. Task-related functional connectivity alterations are suggested to support changes in activations in a task-related and specific manner (39). We have chosen changes in task-related functional connectivity as primary outcome, because it is believed that functional connectivity changes, which resemble synchronization changes, require stronger effects of an intervention than brain activation changes do.

Our results are consistent with a previous study that showed altered activity in similar regions following 4 weeks of consumption of a fermented milk product with probiotics (4). Tillisch et al. were the first to examine the EAT paradigm with regard to possible effects of a probiotic intervention (*Bifidobacterium animalis subsp lactis* administered in a fermented milk product) (4). The main findings of Tillisch et al. were based on network analyses focusing on the examination of the probiotics associated EAT effect in comparison to resting state brain function, rather than the effect of probiotic intervention on the response toward the EAT paradigm itself. They found that the probiotic formula resulted in decreased responses of a widely distributed network of regions involving primary interoceptive and somatosensory regions such as prefrontal cortex, precuneus, basal ganglia, the parahippocampal gyrus as well as a cluster in the midbrain region centered on the periaqueductal gray. This corresponds with the findings of decreased functional connectivity in the present study. However, some of the findings in terms of activity contradict each other. For example, they reported a decreased activity in the frontal regions after probiotic intervention, whereas we found an increased activity in this brain region after probiotic intervention compared to placebo. It should be noted that beneficial effects are not necessarily associated with decreased brain activity as the functional consequence is dependent on the function of the specific brain regions and their interaction.

Most of the brain regions showing increased activity after our probiotic intervention are also involved in cognition. Thus, this study revealed the role of probiotic intervention in increasing activity of regions involved in cognitive processing of emotional stimuli. Similarly, Bagga et al. showed an altered activity in precuneus, midcingulum and parahippocampal gyrus during an emotional decision-making task after a 4-week intervention with a multi-strain probiotic containing nine bacterial strains belonging to *Lactobacillus*, *Lactococcus* and *Bifidobacterium* species in healthy subjects (8). This highlights the concept that brain areas associated with emotional processing are closely connected to brain regions implicated in memory formation and decision making (8) and that probiotics may be able to modulate both.

Different findings between probiotic studies could for example be explained by the usage of different probiotic strains. Species of the *Lactobacilli* and *Bifidobacteria* genera are the most systematically studied probiotics as well as so-called psychobiotics—probiotics that have a positive mental health benefit to the host (40). Animal studies revealed that strains belonging to *Lactobacillus helveticus*, *Lactiplantibacillus plantarum* and *Bifidobacterium longum* have potential to decrease anxiety and depression and enhance memory and social interactions (41). In humans, strains belonging to *Lactobacillus helveticus*, *Bifidobacterium longum* and *Lactiplantibacillus plantarum* have been previously reported to improve psychological symptoms (42–46). The latter has also been shown to improve emotion and attention (46). To date, only a single preclinical study applied a combination of the three species, importantly also using the same strains as in the present study, in a mouse model of chronic mild stress. In this mouse model, the probiotic treatment partially reversed negative behavioral changes (47). Therefore, the psychobiotic properties of a combination of these probiotic species in humans has been tested in the present clinical study for the first time together with our findings during acute stress (48).

Of note is that the probiotic product formulation contained additional bioactive ingredients which could have affected the results. For example, glutathione and lactoferrin are known to possess immunomodulatory activity, which is one potential route of gut-brain communication (49, 50). Magnesium and zinc are essential to many enzymatic and/or regulatory functions influencing both immune and nervous system function (51). The prebiotic inulin has been shown to impact gut microbiota composition and immune function, also potentially influencing the gut-brain axis (52). In the present study a well-nourished study population was selected, and therefore it is unlikely that the additional bioactive ingredients contributed substantially to nutrient intake beyond normal nutrition as the levels of these ingredients in the used formulation were within typical daily intake estimates (53–55). However, it is possible that in less nourished individuals, these ingredients could have a greater impact.

The length of our intervention period was 4 weeks, a typical timeframe for clinical studies assessing probiotic effects. Four weeks have been sufficient to reveal probiotic effects on brain activity and behavior as well as on gastrointestinal symptoms and on small intestinal and colonic level in previous studies (4, 8, 56, 57). Nevertheless, the intervention period could have been too short to capture all effects.

The probiotic intervention had only minimal effects on the gut microbiota composition, analyzed based on the analysis of fecal samples. It is important to note, that this study was not designed and powered to detect subtle microbiota changes. The abundance of the probiotic bacteria contained in our study product was not affected by the intervention; however, on higher taxonomic levels, a small, albeit non-significant, increase of the species *Bifidobacterium longum* and the genera *Bifidobacteria* and *Lactobacillus* could be detected. The fact that the fecal microbiota composition was not majorly altered could indicate that the shown psycho(bio)logical effects are due to microbe-host interactions at the small bowel level, the common habitat of *Lactobacilli*, rather than in the colon. Alternatively, the probiotics could have acted on the colonic ecosystem *via* altered metabolite production or effects on the gut barrier instead. For example, a recently published study showed that the concentrations of the fecal short-chain fatty acids acetate, butyrate and propionate were increased after a 4-week intervention with *Lactiplantibacillus plantarum* in triathletes (58).

Regarding the choice of statistical analyses, we chose to follow an approach that is a compromise between the power of our analyses and the small probability of false positive findings in the result: Spatial smoothing has been performed solely as part of the data preprocessing. Otherwise, no spatial autocorrelation was modeled in any downstream analyses. Likewise, for defining the basic paradigm ME − MS response (applied only to placebo intervention results), subject effects were coded as a fixed effect for a rigid correction of average individual signal level only, keeping the time course of the signal. Results of the basic paradigm ME − MS response were corrected for multiplicity using Bonferroni correction on voxel level and defined based on cluster-forming thresholds on the level of ME − MS activated clusters. Arguably, this comes with a price of limited power regarding small signals. We think, however, that, given the rather small sample size in our study (*n* = 22 subjects), valid small signals could anyhow not reliably be detected.

However, for the analysis of the intervention effects on the level of brain activity (based on the ME − MS activated clusters), we implemented a mixed effects model to allow for more in-depth modeling of individual variations in the emotional responses. The conservative selection of ME − MS activated clusters allowed us to report those results of intervention effects more liberally, both with and without multiplicity correction, in order to facilitate hypothesis generation.

Additionally, the ME − MS activated clusters were used as basis for the downstream functional connectivity analysis. Those results are reported using significance based on FDR estimates, which is the standard procedure in the field.

The findings of the present study are quite subtle, which could be attributed to the rather small sample size of 22. This small sample size is also the reason why subgroup analyses were not possible. Another limitation might be that we did not include fMRI examinations before the intervention periods. We have chosen to do so in order to prevent potential learning effects. However, this study benefited from its crossover design where subjects served as their own controls. We investigated how a probiotic intervention modulated brain activity in young, healthy subjects. Stronger, more robust findings might be detected when studying patients with altered emotional processing, such as patients with anxiety or depression symptoms and are of great interest in order to confirm our findings of dampened negative emotional reactivity. For this very reason, our report of subtle changes in a healthy study group are important, as this further supports the novel concept that probiotic intervention is sufficient to modulate brain activity even in a healthy study population. Additionally, the effect of the probiotic intervention in unchallenged situations, such as daily life, should be further investigated (e.g., based on psychological symptoms). Future studies should thoroughly consider potential effects of a specific probiotic in order to hypothesize and select the appropriate outcome parameters.

In summary, the results of this study revealed that a 4-week intervention with a probiotic mixture containing *Bifidobacterium longum* R0175, *Lactobacillus helveticus* R0052 and *Lactiplantibacillus plantarum* R1012 is indeed associated with changes in brain activity related to, amongst others, emotion. These findings support the assumption that certain probiotics are able to modulate brain function by affecting emotional circuitry in healthy subjects. However, the modes of action of how certain probiotic strains can infer these effects remain to be determined. Future studies should elucidate mechanistic pathways, such as the involvement of metabolites acting directly on the brain or indirectly *via* the vagus nerve.

## Data Availability Statement

The original microbiota sequencing data presented in the study are publicly available. This data can be found here: BioProject: PRJNA789789; http://www.ncbi.nlm.nih.gov/bioproject/789789. Other original data presented in the manuscript will be made available upon request.

## Ethics Statement

The studies involving human participants were reviewed and approved by Ethical Review Board of Uppsala, Sweden (www.etikprovningsmyndigheten.se, registration number: 2017/398 A and B). The patients/participants provided their written informed consent to participate in this study.

## Author Contributions

JR, HEC, JK, DR, PT, LLS, and RB designed research. JR, HEC, and JK conducted research. PT, AK, LLS, AM, and JL provided essential materials. JR, HEC, DR, AH, PT, PA, JP, BS, and RB analyzed data. JR, HEC, JK, DR, AH, PT, JP, BS, and RB wrote the manuscript. RB had primary responsibility for the final manuscript. All authors read and approved the final version of the manuscript.

## Conflict of Interest

LLS was employed by Pfizer Consumer Healthcare. This study received funding from Pfizer Consumer Healthcare. LLS was involved in study design and discussion of the results, but had no involvement in collection, analysis, interpretation of data or the decision to submit this article for publication. The remaining authors declare that the research was conducted in the absence of any commercial or financial relationships that could be construed as a potential conflict of interest.

## Publisher’s Note

All claims expressed in this article are solely those of the authors and do not necessarily represent those of their affiliated organizations, or those of the publisher, the editors and the reviewers. Any product that may be evaluated in this article, or claim that may be made by its manufacturer, is not guaranteed or endorsed by the publisher.

## Footnotes

1
www.etikprovningsmyndigheten.se
