# Supplementary material for: Probiotic Mixture Containing Lactobacillus helveticus, Bifidobacterium longum and Lactiplantibacillus plantarum Affects Brain Responses Toward an Emotional Task in Healthy Subjects: A Randomized Clinical Trial
Source: Front Nutr. 2022 Apr 29;9:827182. doi: 10.3389/fnut.2022.827182 (PMC9104811; doi:10.3389/fnut.2022.827182)
Supplement: Supplementary file 1 [file Data_Sheet_1.pdf]

SUPPLEMENTARY INFORMATION

Probiotic mixture containing *Lactobacillus helveticus*, *Bifidobacterium longum* and *Lactiplantibacillus plantarum* affects brain responses toward an emotional task in healthy subjects: A randomized clinical trial

Julia Rode* and Hanna MT Edebol Carlman*, Julia König, Dirk Repsilber, Ashley N Hutchinson, Per Thunberg, Pernilla Andersson, Jonas Persson, Andrey Kiselev, Lori Lathrop Stern, Benita Salomon, Ahmed Abdulilah Mohammed, Jennifer S Labus, Robert J Brummer.

* Equal contribution/ shared first authorship

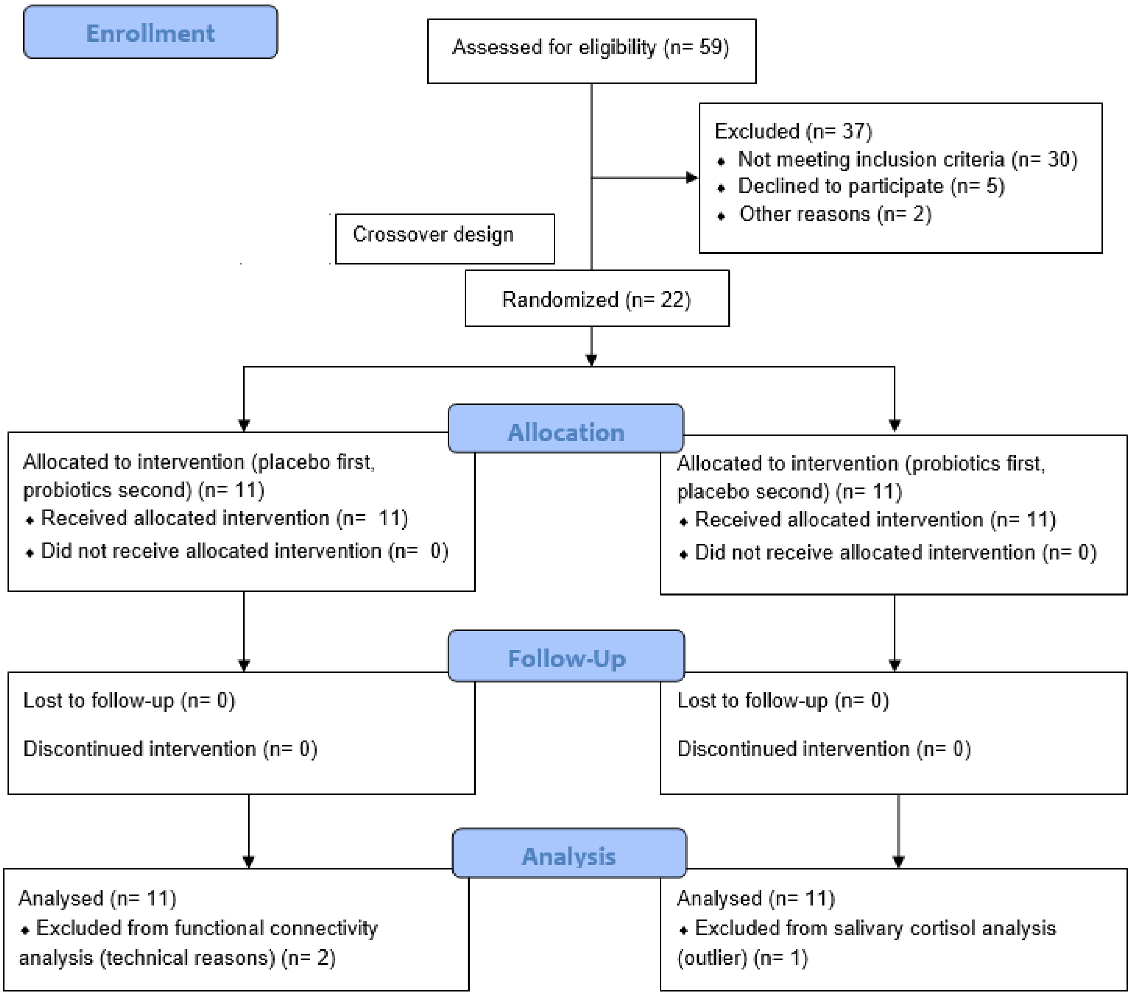

Supplementary Figure 1. Participant flow chart

Supplementary Table 1. Composition of the probiotic product

**Active Ingredients**	**Amount per sachet**
**Inulin**	500 mg
**Magnesium**	45 mg
**Potassium**	50 mg
**Zinc**	9 mg
**Glutathione**	20 mg
**Lactoferrin**	10 mg
**Probiotic blend: *L. helveticus R0052 (CNCM-I-1722), B. longum R0175 (CNCM-I-3470), L. plantarum R1012 (CNCM-I-3736)***	3 x 10^9 CFU

Ingredients:
Fructose, Inulin, Magnesium Gluconate; Acidifying agent: Citric Acid; Aroma, Potassium Citrate, Mixture of live lactic cultures (*Lactobacillus Acidophilus* (*Helveticus*), *Bifidobacterium Longum*, *Lactiplantibacillus Plantarum*), Zinc Gluconate, Magnesium Oxide; Anti-caking agent: Silicon Dioxide; Glutathione; Sweeteners: Acesulfame K, Sucralose; Maltodextrin, Lactoferrin; Dyes: E102, E124.
(underlined ingredients were also contained in the placebo)

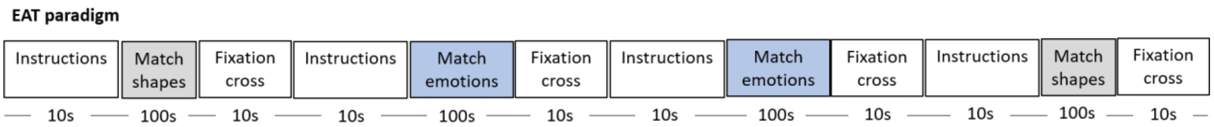

Supplementary Figure 2. Scheme of the Emotional Attention Task (EAT)

Supplementary Information. Preprocessing of fMRI data for activity analysis

Before preprocessing, for any analyses – activity or connectivity – all DICOM images were converted to NIfTI format (using dcm2nii) using SPM12. Furthermore, the origin in structural and fMRI images were all set to the anterior commissure in order to facilitate the preprocessing and to avoid co-registration failures.
For preprocessing prior to the fMRI activity analysis, default settings were applied in the following preprocessing steps (differences from default values within parenthesis):

- Realignment of fMRI images, no wrapping was applied
- Segmentation of the structural image
- Co-registration of structural image and fMRI images. In the co-registration process the structural image was co-registered to the mean of the fMRI images
- Normalization of the fMRI images (voxel size set to 3x3x3 mm^3 which is closer to the original resolution of the acquired images) to MNI space
- Normalization of the structural image (voxel size set to 1x1x3 mm^3 which matched the original resolution) to the MNI space
- Smoothing (kernel = 6 mm in order to increase the specificity while losing some sensitivity, thus leading to a more conservative approach)

For each subject a simple 1st-level analysis was performed which served as a sanity check and where a typical pattern was observed using the rough contrast ME>MS. This was done to control for proper preprocessing of the data and that reasonable activations could be observed by the paradigm itself independent of any interventional effect.
A script was written which performed conversion to NIfTI file format, preprocessing and sanity check. This was to ensure that all examinations were equally preprocessed prior to the following group analysis.

Supplementary Table 2. Brain regions with relevance in literature
All brain regions that have been selected based on previous literature are presented. Those pre-defined regions that were further significantly activated in the task (ME>MS) after the placebo intervention are defined as regions of interest and are marked italic. Multiple comparison correction with Bonferroni. Significance level $p<0.05/246$.

**Anatomical region**	**BNA region**	**ME – MS effect size**	**BNA region**	**ME – MS effect size**
Precueneus, area 31	*153*	*1.790*	*154*	*2.619*
Medial amygdala	*211*	*1.653*	*212*	*1.723*
Lateral amygdala	*213*	*1.608*	*214*	*2.118*
Rostral temporal thalamus	*237*	*3.262*	*238*	*3.134*
Rostral hippocampus	*215*	*0.692*	*216*	*0.865*
Parahippocampal gyrus, rostral area 35/36	*109*	*1.015*	*110*	*0.985*
Parahippocampal gyrus, area 28/34	115	-0.271	116	-0.050
Globus pallidus	*221*	*0.869*	*222*	*1.600*
Ventral caudate	*219*	*2.261*	*220*	*1.901*
Ventromedial putamen	*225*	*1.727*	*226*	*2.156*
Dorsolateral putamen	229	0.301	*230*	*1.388*
Cingulate gyrus, pregenual area 32	*179*	*1.431*	*180*	*1.944*
Cingulate gyrus, subgenual area 32	187	-1.611	188	-0.253
Cingulate gyrus, rostraventral area 32	*177*	*1.480*	178	-0.126
Superior temporal gyrus, caudal area 22	*75*	*1.704*	*76*	*2.429*
Superior temporal gyrus, rostral area 22	*79*	*1.387*	*80*	*3.097*
Medial prefrontal cortex, median area 9	*11*	*3.736*	*12*	*3.194*
Medial prefrontal cortex, median area 10	*13*	*3.774*	*14*	*5.478*
Dorsolateral prefrontal cortex, lateral area 9	*5*	*5.310*	*6*	*3.543*
Dorsolateral prefrontal cortex, area 46	*19*	*3.766*	*20*	*2.279*
Orbitofrontal cortex, orbital area 12/47	*43*	*2.593*	*44*	*4.033*
Orbitofrontal cortex, lateral 11	45	0.550	*46*	*1.789*
Orbitofrontal cortex, medial area 11	*47*	*2.016*	*48*	*2.331*
Primary somatosensory cortex, area 2	159	0.305	*160*	*1.082*
Primary somatosensory cortex, dorsolateral area 6	7	-0.111	8	0.255
Primary somatosensory cortex, medial area 6	9	0.035	10	-0.087
Frontal cortex, dorsolateral area 8	3	0.381	*4*	*0.848*
Frontal cortex, ventral area 44	*39*	*3.794*	*40*	*5.297*
Insular cortex, ventral angular insula	*165*	*3.105*	*166*	*3.473*
Insular cortex, dorsal granular insula	171	-0.408	*172*	*1.208*

BNA – Brainnetome atlas; ME – Match emotions; MS – Match shapes

Supplementary Table 3. Clusters

Clusters that were activated by the task (ME>MS) after the placebo intervention are presented. The selected regions of interest (defined by previous literature and their involvement in the task) are marked italic. Clusters that spanned over several BNA regions were divided into sub-clusters. Multiple comparison correction with Bonferroni.

**MNI coordinates of peak (x y z)**	**Cluster size [mm^3]**	**ME – MS effect size**	**Anatomical region**
48 45 -15	*378*	*6.712*	*Orbital gyrus, A12/47o*
-30 21 0	*8343*	*5.880*	*Dorsal agranular insula*
21 -6 -12	*837*	*3.827*	*Medial amygdala*
-21 -12 -9	405	4.708	Medial amygdala
30 -27 -3	1269	3.360	Caudal hippocampus
6 66 6	2673	10.241	Medial area 10
6 6 3	270	4.697	Dorsal caudate
-9 9 6	675	4.274	Dorsal caudate
-48 -51 9	594	3.899	Caudoposterior superior temporal sulcus
-45 15 24	14472	6.721	Dorsal area 44
-21 69 15	756	8.414	Lateral area 10
12 9 15	*297*	*3.863*	*Dorsal caudate*
-9 63 21	2862	10.319	Medial area 10
9 -63 36	*1350*	*4.417*	*A31, area 21 (Lcl)*
36 -54 45	5265	4.867	Intraparietal area 7 (hIP3)
-33 -57 45	3861	5.367	Rostrodorsal area 39 (hIP3)
-3 27 42	3213	4.910	Medial area 9
Large cluster divided into sub-clusters:			
51 -27 0	459	3.849	Rostral area 22
66 -42 -9	648	4.601	Caudal area 21
57 -48 6	2430	6.036	Dorsolateral area 37
60 -6 -15	1917	4.016	Anterior superior temporal sulcus
51 -57 -21	351	5.102	Extreme lateroventral area 37
63 -42 -12	432	4.906	Caudolateral of area 20
39 -60 -18	5427	11.509	Medioventral area 37
42 -51 -21	4644	10.310	Lateroventral area 37
51 -42 6	1539	5.125	Rostroposterior superior temporal sulcus
51 -42 9	945	6.338	Caudoposterior superior temporal sulcus
54 -66 15	*1026*	*5.265*	*Caudal area 39 (PGp)*
48 -45 12	1755	5.321	Rostroventral area 39 (Pga)
21 -93 -3	1539	7.448	Caudal lingual gyrus
24 -66 -3	1350	5.707	Rostral lingual gyrus
39 -78 0	2511	6.130	Middle occipital gyrus
42 -72 -6	1971	8.947	Area V5/MT+
24 -93 0	2106	6.727	Occipital polar cortex
42 -78 -12	5427	12.558	Inferior occipital gyrus
Large cluster divided into sub-clusters:			
-39 -75 -15	5157	12.058	Medioventral area 37

-39 -51 -18	4293	11.485	Lateroventral area 37
-21 -78 -6	1431	7.966	Caudal lingual gyrus
-30 -93 6	1323	5.693	Middle occipital gyrus
-42 -75 -6	675	8.470	Area V5/MT+
-18 -93 -6	1107	5.144	Occipital polar cortex
-39 -78 -12	*3888*	*12.113*	*Inferior occipital gyrus*
Large cluster divided into sub-clusters:			
54 21 30	3051	6.866	Inferior frontal junction
45 27 21	648	6.722	Ventral area 9/46
54 27 30	1215	8.094	Ventrolateral area 8
54 21 27	2268	9.597	Dorsal area 44
51 30 21	1107	8.387	Inferior frontal sulcus
57 24 21	1134	7.423	Caudal area 45
54 42 -3	837	6.648	Rostral area 45
51 24 -3	945	5.589	Opercular area 44
48 15 18	837	6.545	Ventral area 44
36 36 -9	*432*	*4.692*	*Orbital area 12/47*
42 27 -9	*1188*	*5.906*	*Lateral area 12/47*
42 9 30	3402	7.561	Caudal ventrolateral area 6
36 24 0	*810*	*6.117*	*Dorsal agranular insula*

BNA – Brainnetome atlas; ME – Match emotions; MS – Match shapes; MNI – Montreal Neurological Institute

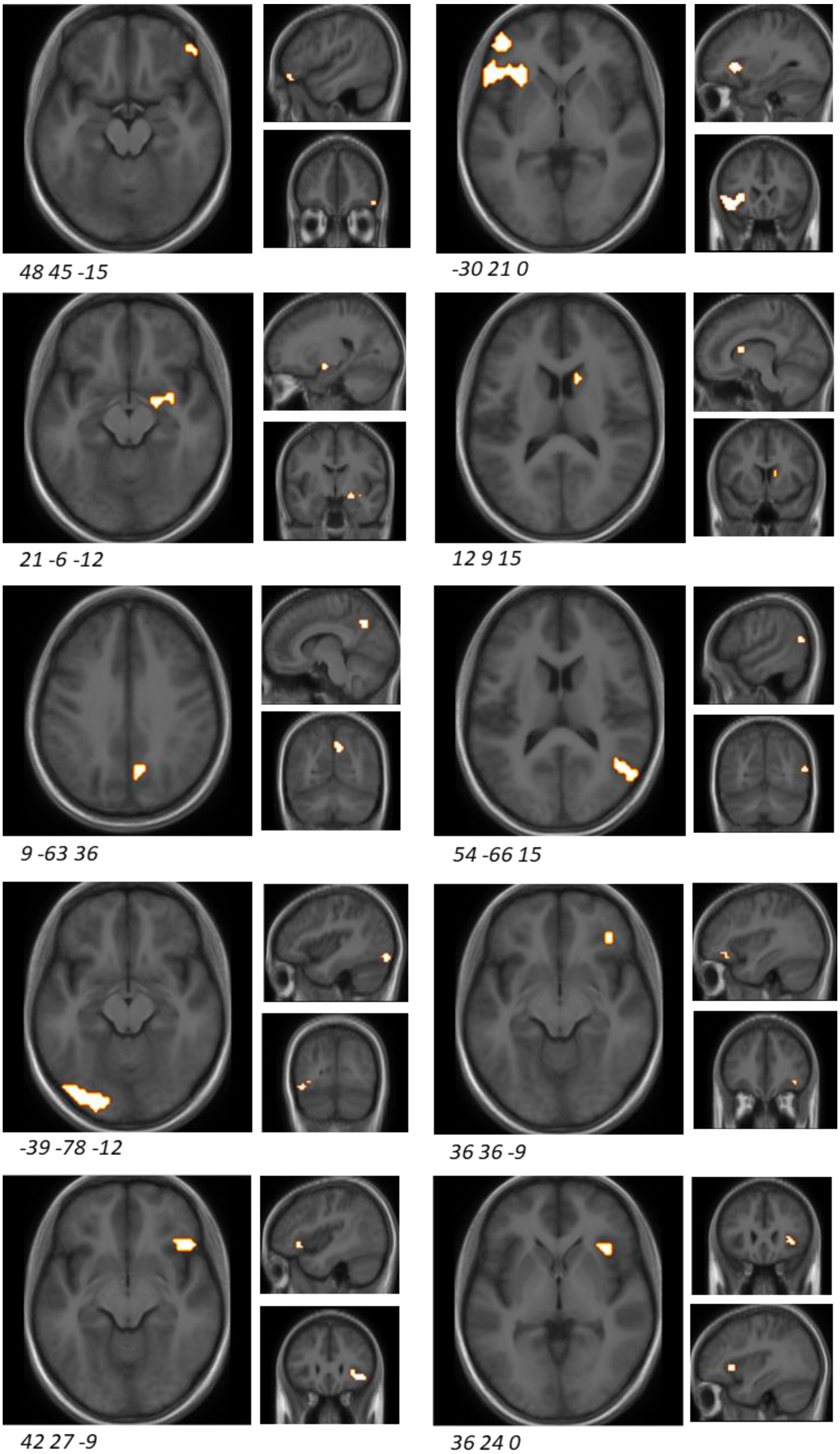

Supplementary Figure 3. Clusters selected based on their *a priori* relevance in the literature and due to their involvement in the task

Defined as regions of interest. Involvement in the task has been examined as ME – MS contrast after the placebo intervention only. Clusters are superimposed on average anatomical scans. Clusters can be identified by the coordinates of their peak (x y z).

Supplementary Table 4. Baseline characteristics
The baseline characteristics did not differ significantly between the two study arms (1. probiotics & 2. placebo vs. 1. placebo & 2. probiotics).

**Age**	24.2 ± 3.4 years
**Male/female**	6/16
**BMI***	22.3 kg/m^2 (21.2 to 23.8)
**Hospital Anxiety and Depression Scale***	
• **Total score**	5.0 (4.0 to 8.0)
• **Depression subscore**	1.0 (0.8 to 2.0)
• **Anxiety subscore**	4.0 (2.0 to 6.3)
**State and Trait Anxiety Inventory***	
• **State subscore**	26.0 (22.0 to 30.5)
• **Trait subscore**	28.0 (25.0 to 33.3)
**Perceived Stress Scale (total score)***	9.0 (5.8 to 12.3)
**Quality of life (based on EQ-5D-5L index value)***	80.5% (75.0 to 90.0)

*For BMI and all questionnaire scores median and interquartile range (IQR 25 to 75) are reported
BMI – Body mass index

Supplementary Table 5. Baseline diet
Subjects were asked to fill in a 3-day food diary. The three days have been analyzed separately, but average nutrients of the three days are reported. Median and IQR (25 to 75) are reported. The background diet of all subjects of the two study arms (1. probiotics & 2. placebo vs. 1. placebo & 2. probiotics) did not differ significantly.

Protein [g]	Fat [g]	Carbohydrates [g]	Alcohol [g]	Fibers [g]	Water [g]
84.0 (72.0 to 90.1)	107.7 (90.3 to 117.5)	210.2 (182.5 to 235.6)	0.0 (0.0 to 0.0)	23.5 (18.6 to 27.8)	1848.0 (1554.0 to 2055.0)

Supplementary Table 6. Physical activity
Physical activity was recorded during one week before the start and during the last week of each intervention period by Actigraphy (Actiwatch spectrum-pro and Actiware 6.0 software, Philips Respironics, Andover, MA, USA) and averaged per week. Physical activity did not differ significantly between the two study arms nor changed significantly during the course of the study. Median and interquartile range (IQR 25 to 75) are reported.

One week at 1. baseline [counts/ minute]	Last week of 1. intervention [counts/ minute]	One week at 2. baseline [counts/ minute]	Last week of 2. intervention [counts/ minute]
188.2 (146.2 to 218.9)	185.0 (143.2 to 211.9)	182.4 (146.5 to 214.2)	183.0 (153.8 to 261.2)

Supplementary Table 7. Adverse events

The total number of adverse events is reported, the number of those events which were suspected to be related to the intervention or possible related to the intervention are shown in brackets: total number (suspected/possible).

Symptom	Placebo	4 weeks after placebo	Probiotic	4 weeks after probiotic
**Abdominal pain**	2 (0/2)	0 (0/0)	1 (0/1)	0 (0/0)
**Diarrhea**	0 (0/0)	1 (0/0)	2 (0/2)	0 (0/0)
**Bloating**	1 (0/1)	0 (0/0)	1 (0/1)	0 (0/0)
**Nausea**	1 (0/1)	0 (0/0)	2 (0/2)	1 (0/0)
**Headache**	12 (4/1)	1 (0/0)	5 (0/2)	0 (0/0)
**Cold**	14 (0/0)	0 (0/0)	6 (0/1)	3 (0/0)
**Sore throat**	1 (0/0)	0 (0/0)	0 (0/0)	1 (0/0)
**Fever**	1 (0/0)	0 (0/0)	1 (0/0)	0 (0/0)
**Sleep problems**	2 (0/2)	0 (0/0)	1 (0/1)	0 (0/0)
**Depressive symptoms**	0 (0/0)	1 (0/1)	0 (0/0)	1 (0/1)
**Anxiety symptoms**	0 (0/0)	0 (0/0)	1 (0/1)	0 (0/0)
**Pain in the knee**	0 (0/0)	0 (0/0)	2 (0/0)	0 (0/0)
**Fungal infection**	1 (0/1)	0 (0/0)	0 (0/0)	0 (0/0)
**Pollen allergy**	1 (0/0)	0 (0/0)	4 (0/0)	0 (0/0)

Supplementary Table 8. Compliance by intervention period

All subjects consumed 90% or more of the study product.

	Daily consumption of the study product	One single day consumption missed during second intervention	One day consumption missed in both interventions	Several days consumption missed during first intervention and one day missed during second intervention
**Number of subjects**	14 (63.6%)	4 (18.2%)	2 (9.1%)	2 (9.1%)*

*Two subjects forgot to consume the intervention product on 3 and 4 days, respectively, during the first intervention period, with a maximum of two missing consecutive days.

Supplementary Table 9. Compliance by product

All subjects consumed 90% or more of the study product.

	Daily consumption of the study product	One single day consumption missed	Several days consumption missed
**Placebo**	14 (63.6%)	8 (36.4%)	0 (0.0%)
**Probiotics**	18 (81.8%)	2 (9.1%)	2 (9.1%)*

*Two subjects forgot to consume the intervention product on 3 and 4 days, respectively, with a maximum of two missing consecutive days.

Supplementary Information. Whole-brain analysis based on BNA regions

Additionally to a cluster-based analysis focusing on functionally altered areas a whole-brain analysis was performed based on anatomically defined brain regions. The intensity of signals of all voxels of individual brainnetome atlas (BNA) regions were averaged per brain volume, keeping the time course of the signal. The regions of significant p-values were defined using the BNA atlas. Among those regions being involved in the task, n=26 overlapped with the literature-based selection and were selected for the region-based analysis (Supplementary Table 2) investigating the intervention effect.

fMRI activity profiles for BNA regions were computed as averages of the composing voxels prior to modeling them according to equation 1 and 2 in the same way as done for the cluster-based analysis.

Comparing the probiotic and the placebo intervention, BNA region-based analysis showed significant ($p<0.1$) changes in activation (ME versus MS) in several regions that were among the predefined regions of interest (without correction for multiplicity) (Supplementary Table 10). Reduced activation (probiotics < placebo) was observed in the parahippocampal gyrus, hippocampus, amygdala, basal ganglia and cingulate gyrus. Increased activation (probiotics > placebo) was seen in the orbital gyrus and precuneus.

Supplementary Table 10. BNA regions that were found to be associated with significant ($p<0.1$) changes in brain activity between both interventions before multiplicity correction, during the EAT paradigm. Eleven of these regions were predefined regions of interest (italic).

**BNA region**	**ME – MS in probiotic – placebo p-value***	**ME – MS in probiotic – placebo effect size**	**Anatomical region**
115	*0.006*	*-1.140*	*Parahippocampal gyrus, area 28/34*
30	0.007	2.014	Inferior frontal gyrus, dorsal area 44
29	0.012	2.080	Inferior frontal gyrus, dorsal area 44
52	*0.018*	*2.124*	*Orbital gyrus, lateral area 12/47*
220	*0.026*	*-2.150*	*Basal ganglia, ventral caudate*
213	*0.030*	*-2.098*	*Amygdala, lateral amygdala*
221	*0.037*	*-0.948*	*Basal ganglia, globus pallidus*
149	*0.046*	*0.914*	*Precuneus, medial area 5*
81	0.046	1.075	Middle temporal gyrus, caudal area 21
223	0.050	-1.421	Basal ganglia, nucleus accumbens
199	0.057	-1.190	Lateral occipital cortex, middle occipital gyrus
117	*0.069*	*-1.240*	*Parahippocampal gyrus, area TI*
215	*0.075*	*-1.390*	*Hippocampus, rostral*
64	0.075	1.429	Precentral gyrus, caudal ventrolateral area 6
87	0.081	0.597	Middle temporal gyrus, anterior superior sulcus
219	*0.085*	*-1.274*	*Basal ganglia, ventral caudate*
211	*0.088*	*-1.320*	*Amygdala, medial*
178	*0.096*	*-1.343*	*Cingulate gyrus, rostroventral area 24*

*After correction for multiple testing using Bonferroni, the changes in activation in all BNA regions ($p>0.05/246$) and the predefined ROIs ($p>0.05/26$) did not remain statistically significant.

BNA – Brainnetome atlas, EAT – Emotional Attention Task, ME – Match emotions, MS – Match shapes
